# Postpartum Body Concerns, Self-Criticism, and Psychological Distress: A Parallel Mediation Analysis

**DOI:** 10.3390/jcm15176912

**Published:** 2026-09-07

**Authors:** Ana Beato, Cristiana C. Marques, Catarina Ribeiro, Stephanie Alves, Sara Albuquerque

**Affiliations:** 1 HEI-Lab: Digital Human-Environment Interaction Labs, Lusófona University, 1749-024 Lisbon, Portugal; cristiana.marques@ulusofona.pt (C.C.M.); stephanie.alves@ulusofona.pt (S.A.); sara.albuquerque@ulusofona.pt (S.A.); 2 School of Psychology and Life Sciences, Lusófona University, 1749-024 Lisbon, Portugal; cataribeiro04@gmail.com

**Keywords:** postpartum body concerns, self-criticism, self-reassurance, depression, anxiety, stress, maternal mental health

## Abstract

**Background**: Postpartum body concerns have been consistently associated with greater maternal psychological distress, although the psychological processes associated with this relationship remain insufficiently understood. This study examined whether self-criticism (inadequate self and hated self) and self-reassurance mediate the association between postpartum body concerns and symptoms of depression, anxiety, and stress. **Methods**: A cross-sectional study was conducted with 540 Portuguese mothers of infants aged 2 weeks to 24 months. Participants completed measures of postpartum body concerns, the Forms of Self-Criticizing and Self-Reassuring Scale (FSCRS), and Depression, Anxiety and Stress Scales–21 Items (DASS-21). Parallel mediation analyses with bootstrap estimation (5000 samples) were performed. **Results**: Greater postpartum body concerns were associated with higher depression, anxiety, and stress, as well as greater self-criticism and lower self-reassurance. Significant indirect associations involving self-relating styles were observed for depressive, anxiety, and stress symptoms. For anxiety and stress, the direct associations between postpartum body concerns and symptoms were no longer statistically significant after accounting for the three mediators. Inadequate self showed the strongest indirect associations, particularly for stress, while hated self and self-reassurance also showed significant indirect associations across all three models. **Conclusions**: Self-criticism and self-reassurance may represent candidate psychological processes involved in the associations between postpartum body concerns and psychological symptoms. Longitudinal research is needed to further examine their potential role in postpartum psychological adjustment.

## 1. Introduction

Pregnancy and the postpartum period involve substantial physical, hormonal, and psychosocial changes, which may enhance vulnerability to emotional difficulties [1], putting women at increased risk for elevated levels of depression, anxiety, and stress [2]. Globally, prevalence estimates indicate that postpartum depression affects approximately 17% of women [3], while postpartum anxiety affects around 12% [4], and comorbid anxiety and depression symptoms occur in about 8% of postpartum women [5]. Among the factors linked to maternal adjustment, body image has received growing attention due to the significant bodily changes that occur during the perinatal period [6].

Body image refers to individuals’ perceptions, thoughts, emotions, and attitudes toward their bodies [7]. During pregnancy and the postpartum period, physical changes may challenge body satisfaction, particularly in sociocultural contexts that value thinness and rapid restoration of the pre-pregnancy appearance [8]. Accordingly, body dissatisfaction has been consistently associated with higher levels of depressive, anxiety, and stress symptoms among perinatal women [9,10].

The postpartum period may be especially relevant for understanding these associations. Many women report concerns about weight retention, changes in body shape, and other lasting physical alterations after childbirth [8,10]. At the same time, social and media messages may promote unrealistic expectations regarding postpartum recovery and the rapid restoration of a pre-pregnancy body, contributing to appearance-related pressures and social comparison [11]. Recent research applying sociocultural models of body image in the perinatal period suggests that appearance pressures from media, family, and significant others may contribute to body dissatisfaction through appearance comparison and internalization of appearance ideals [12]. When such appearance ideals are internalized, women may evaluate their changing bodies against culturally valued standards and perceive a discrepancy between their actual physical appearance and idealized appearance. These perceived discrepancies may extend body concerns beyond dissatisfaction with physical changes to broader evaluations of personal inadequacy and failure. This process may be particularly relevant during the transition to motherhood, when body-related evaluations occur alongside major identity changes, new caregiving responsibilities, sleep disruption, and relationship adjustments [13].

Although the association between body dissatisfaction and postpartum psychological distress is well established, less is known about the processes that may account for this relationship. One candidate mechanism is self-criticism, which has been identified as a vulnerability factor for several forms of psychopathology, particularly depression and stress-related difficulties [14,15,16]. Negative evaluations of postpartum bodily changes may activate self-critical cognitive-affective processes, particularly when women perceive themselves as failing to meet internalized societal expectations regarding appearance and recovery after childbirth [17]. In this context, self-criticism may represent one pathway through which postpartum body concerns become associated with psychological distress.

According to Gilbert et al. [18], self-criticism is not a unitary construct. The inadequate self-dimension reflects feelings of inferiority, failure, and personal inadequacy. The hated self-dimension represents a more hostile form of self-relating characterized by self-directed anger, rejection, and self-attack. In contrast, reassured self reflects the ability to respond to setbacks with understanding, encouragement, and self-compassion. These distinct forms of self-relating may represent different psychological pathways associated with postpartum body concerns and psychological distress. Women who report greater dissatisfaction with postpartum bodily changes may also report stronger feelings of inadequacy when they perceive themselves as falling short of personal or societal expectations [17,19]. For some women, body dissatisfaction may also be associated with more hostile forms of self-evaluation [20]. In contrast, the ability to adopt a reassuring and compassionate stance toward oneself may be associated with lower levels of psychological and emotional distress in the context of these concerns [21]. Examining these dimensions separately may therefore help clarify how postpartum body concerns are associated with psychological distress. Despite growing interest in maternal body image, little is known about the role of specific forms of self-relating in the association between postpartum body concerns and psychological distress [22]. The present study extends previous research by moving beyond the established association between postpartum body concerns and psychological distress to examine whether distinct self-relating styles are statistically involved in these associations. Specifically, inadequate self, hated self, and reassured self were simultaneously examined as parallel mediators of the associations between postpartum body concerns and three indicators of psychological distress: depression, anxiety, and stress. This approach allows for the examination of the unique indirect associations associated with different forms of self-criticism and self-reassurance within the same statistical models, providing a more differentiated account of the psychological processes associated with postpartum body concerns.

Therefore, the present study examined whether self-criticism and self-reassurance statistically accounted for indirect associations between postpartum body concerns and psychological distress (depression, anxiety, and stress). Specifically, the three forms of self-relating—inadequate self, hated self, and reassured self—were examined as parallel mediators. It was hypothesized that higher postpartum body concerns would be associated with higher levels of depressive, anxiety, and stress symptoms, and that these associations would show significant indirect associations involving self-relating styles, particularly inadequate self.

## 2. Materials and Methods

### 2.1. Participants

The sample consisted of 540 Portuguese mothers aged 20 to 48 years (M = 32.77, SD = 5.00), whose infants were aged 0.5 to 24 months (M = 8.59, SD = 6.51). Most participants were married or cohabiting with their partner (90.9%) and had completed higher education (61.9%). The majority were employed or working students (84.3%), and approximately half reported a monthly household income between €1125 and €2291 (52.9%). Regarding obstetric characteristics, 63.5% of participants were primiparous, and 57.0% reported at least one pregnancy-related complication. Vaginal delivery was reported by 51.9% of mothers, while 36.5% reported cesarean delivery, either planned or emergency. Most infants were born at term (93.9%). At the time of assessment, 43.0% of mothers were on parental leave, whereas 57.0% were not. Most participants reported no current maternal health problems (84.3%) (Table 1).

### 2.2. Procedure

This study is part of a broader research project examining risk and protective factors associated with maternal adjustment. Data were collected between February and March 2020, following approval from the Ethics and Deontology Committee of the Psychology and Life Sciences at Lusófona University. Eligibility criteria required participants to be biological mothers of an infant younger than or equal to 24 months old, be aged 18 years or older, have conceived the child within a heterosexual relationship, and have sufficient Portuguese language proficiency to complete the assessment protocol. This criterion was not specific to the research question addressed in the present study but reflected the eligibility framework defined for the broader project. We acknowledge that this criterion restricts the diversity of relationship and family configurations represented in the sample. Participants were recruited through non-probabilistic snowball sampling. Data collection was conducted remotely using the Typeform digital platform, which enabled anonymous participation and facilitated access to mothers from different geographical regions of Portugal. The survey link was disseminated through Facebook groups for mothers, online maternal support communities, and parenting forums.

Before accessing the questionnaires, participants were presented with an electronic informed consent form containing detailed information regarding the aims of the study, confidentiality and anonymity procedures, voluntary participation, data protection measures, the right to withdraw at any moment without consequences, and relevant ethical considerations. Contact information for the research team and mental health support services were also provided in case participation generated emotional discomfort. Participants did not receive any financial compensation for their participation in the study.

A total of 591 participants provided informed consent and were granted access to the assessment protocol (*n* = 591). Forty-one mothers were excluded for incomplete questionnaire responses. Ten additional participants had incomplete data on one or more sociodemographic and obstetric variables included in the adjusted mediation models and were therefore excluded, resulting in an analytic sample of 540 mothers.

An a priori power analysis was conducted using G*Power 3.1 for a fixed-effects multiple regression model (R^2^ increase), with three tested predictors (the parallel mediators) and nine predictors in total (postpartum body concerns, the three mediators, and the five covariates). Assuming a medium effect size (f^2^ = 0.15), an alpha level of 0.0167 to account for the three primary outcomes, and 95% power, the analysis indicated a minimum required sample size of 146 participants. The final sample of 540 participants therefore substantially exceeded the required sample size.

### 2.3. Measures

**Sociodemographic and Risk Factors Questionnaire**

A Sociodemographic and Risk Factors Questionnaire was developed to obtain information regarding participants’ demographic, obstetric, clinical, and psychosocial characteristics, as well as potential risk factors for maternal maladjustment during the perinatal period. The questionnaire included items assessing maternal age, educational level, marital status, employment status, and gestational complications.

**Postpartum Body Concerns**

A composite variable assessing two specific aspects of postpartum body concerns—current body satisfaction and concern about persistent body changes following pregnancy—was derived from two items of the Sociodemographic and Risk Factors Questionnaire. The first item, “Currently, I am satisfied with my body”, assessed current body satisfaction, while the second, “I am afraid that my body will not return to how it was before pregnancy”, assessed concerns about persistent changes relative to the pre-pregnancy body. The first item was reverse-coded so that higher scores on both items reflected greater postpartum body concerns. The two items were combined to provide a brief indicator of postpartum body concerns. This composite was operationalized specifically to capture these two aspects and was not intended to represent the broader, multidimensional construct of postpartum body image. The first item was reverse-coded so that higher scores on both items consistently reflected greater postpartum body concerns. Responses were rated on a 5-point Likert scale, with higher scores indicating greater body concerns (i.e., lower satisfaction with postpartum body image). The composite score was calculated as the mean of the two items, yielding a continuous score ranging from 1 to 5. The correlation between the two items was strong according to Cohen’s [23] criteria (r = 0.58, *p* < 0.001). The Spearman–Brown coefficient was 0.73, indicating satisfactory internal consistency for this two-item composite. Because the two items assess conceptually related, although distinct, aspects of postpartum body-related concerns—namely, current satisfaction with one’s body and concern about persistent changes relative to the pre-pregnancy body—they were combined to provide a brief indicator of postpartum body concerns. The observed inter-item correlation (r = 0.58, *p* < 0.001) and Spearman–Brown coefficient supported the use of the two items as a composite measure. However, these indices provide evidence of internal consistency only and do not establish construct validity. Accordingly, the composite should be interpreted as a brief measure of specific postpartum body concerns rather than as a comprehensive measure of the broader, multidimensional construct of postpartum body image or body dissatisfaction.

**The Forms of Self-Criticizing and Self-Reassuring Scale (FSCRS)**

Self-criticism and self-reassurance were assessed using the Portuguese version of the FSCRS [18,24]. This 21-item self-report instrument evaluates the ways individuals relate to themselves in situations of failure, inadequacy, or personal difficulties. The FSCRS comprises three subscales: Inadequate Self, which assesses feelings of inferiority and inadequacy; Hated Self, which evaluates hostile, punitive, self-directed attitudes; and Reassured Self, which measures the ability to respond to oneself in a compassionate and supportive manner. Items are rated on a 5-point Likert scale ranging from 0 (“Not at all like me”) to 4 (“Extremely like me”). A score for each subscale is computed by averaging the items. Higher scores indicate greater endorsement of the corresponding self-relating style. The FSCRS demonstrated good to excellent internal consistency in the present sample. Cronbach’s alpha coefficients were 0.90 for the Inadequate Self, 0.82 for the Reassured Self, and 0.74 for the Hated Self.

**Depression Anxiety Stress Scales–21 (DASS-21)**

Symptoms of depression, anxiety, and stress were measured using the Portuguese version of DASS-21 [25,26]. DASS-21 comprises 21 items divided into three subscales assessing depressive symptoms, anxiety symptoms, and stress-related symptoms. DASS-21 assesses symptoms of depression, anxiety, and stress and is not a diagnostic measure of psychiatric disorders. Participants rated the extent to which each symptom applied to them during the previous week using a 4-point scale ranging from 0 (“Did not apply to me at all”) to 3 (“Applied to me very much, or most of the time”). A score for each subscale is computed by summing the items (possible range 0–21). Higher scores indicate higher levels of psychological distress. The internal consistency of the DASS-21 subscales was good to excellent in the present sample, with Cronbach’s alpha coefficients of 0.88 for depression, 0.82 for anxiety, and 0.90 for stress.

### 2.4. Data Analysis

Statistical analyses were conducted using IBM SPSS Statistics, version 31.0 (IBM Corp., Armonk, NY, USA), and JASP, version 0.96.0.0 (JASP Team, Department of Psychological Methods, University of Amsterdam, Amsterdam, The Netherlands). Preliminary analyses included descriptive statistics for all sociodemographic and study variables, as well as reliability analyses of the measures used in the study. Internal consistency was assessed using Cronbach’s alpha coefficients. The distributions of the study variables were examined using skewness and kurtosis statistics. Correlational analyses were conducted to explore associations between body concerns, self-relating styles, and psychological distress.

For the regression models estimated as part of the mediation analyses, multicollinearity was assessed using tolerance and variance inflation factor (VIF) statistics. Tolerance values below 0.10 and VIF values above 10 were considered indicative of potentially problematic multicollinearity. To examine the hypothesized indirect associations between postpartum body concerns and psychological distress through self-criticism and self-reassurance, three parallel mediation models were estimated using the PROCESS Macro (Model 4; [27]). In each model, postpartum body concerns were entered as the independent variable, and symptoms of depression, anxiety, and stress were examined separately as dependent variables. The three forms of self-relating (Inadequate Self, Hated Self, and Reassured Self) were included simultaneously as parallel mediators. Based on their theoretical and potential empirical relevance to maternal psychological distress during the postpartum period, maternal age, parity, postpartum duration, maternity leave status (yes/no), and gestational complications (yes/no) were included as covariates in all mediation models. These covariates were entered simultaneously in both the mediator models and the outcome model, such that the estimated associations between postpartum body concerns, self-relating styles, and psychological distress were adjusted for these maternal and obstetric characteristics.

For each model, the total effect of postpartum body concerns on the outcome (c), the direct effect after accounting for the mediators (c′), and the specific indirect effects through each mediator were estimated. Indirect effects were estimated using a bootstrap procedure with 5000 non-parametric bootstrap resamples. An indirect effect was considered significant when the 95% bias-corrected confidence intervals (CIs) did not include zero. In addition, pairwise contrasts between the specific indirect effects were examined to determine whether the magnitude of the indirect association differed significantly across Inadequate Self, Hated Self, and Reassured Self. Statistical significance was set as *p* < 0.05 for all analyses.

## 3. Results

### 3.1. Preliminary Analyses

Most variables showed relatively small deviations in skewness and kurtosis. Greater positive skewness and kurtosis were observed for Hated Self (skewness = 3.31, kurtosis = 12.93), depression symptoms (skewness = 2.14, kurtosis = 5.33), and anxiety symptoms (skewness = 2.50, kurtosis = 7.85).

Descriptive statistics and bivariate correlations between main study variables are presented in Table 2. Higher postpartum body concerns were significantly associated with higher levels of both forms of self-criticism, depression, anxiety, stress, and lower levels of Reassured Self. As expected, the two self-critical dimensions were positively associated with psychological distress, whereas the Reassured Self was negatively associated with both self-criticism and symptoms of depression, anxiety, and stress (all *p* < 0.001).

### 3.2. Mediation Analyses

The results of the parallel mediation analyses are presented in Table 3. No evidence of problematic multicollinearity was identified across the three models, with VIF values ranging from 1.03 to 2.11 and tolerance values ranging from 0.48 to 0.97.

When depression symptoms were considered as the outcome, both the direct and total effects of postpartum body concerns on depressive symptoms were significant. Significant indirect effects were observed through Inadequate Self, Hated Self, and Reassured Self, indicating that greater postpartum body concerns were associated with higher depressive symptoms through each of these self-relating styles, while a significant direct association remained after accounting for the mediators and covariates. The model explained 47.7% of the variance in depressive symptoms.

Although postpartum body concerns were significantly associated with both anxiety and stress at the total-effect level, the corresponding direct effects were no longer statistically significant after accounting for Inadequate Self, Hated Self, and Reassured Self. Significant indirect effects were observed through all three mediators for both anxiety and stress, indicating that the associations between postpartum body concerns and these psychological outcomes were statistically accounted for by indirect effects through self-relating styles.

**Figure 1:**
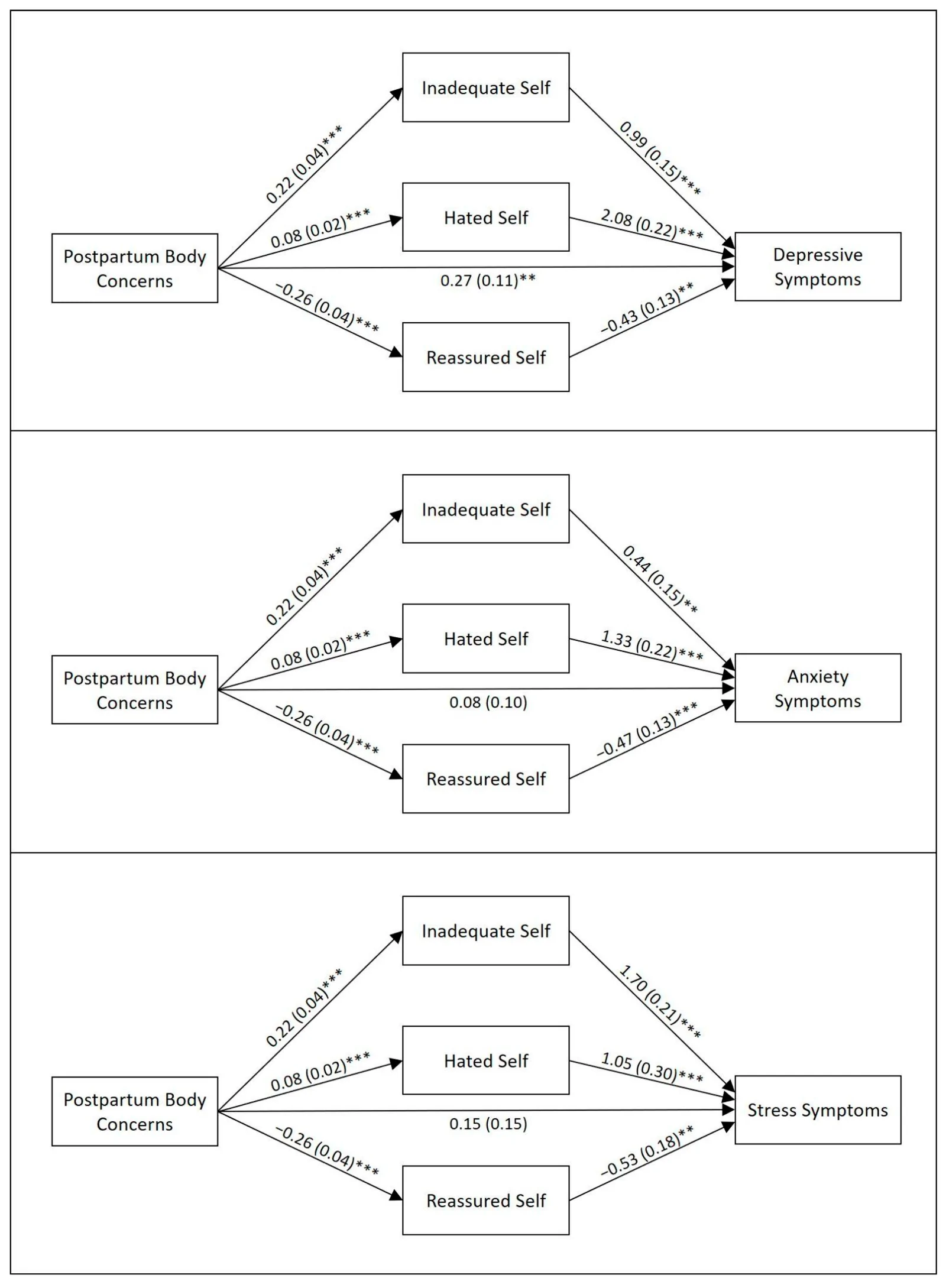
Parallel mediation models examining the associations between postpartum body concerns and depressive symptoms, anxiety symptoms, and stress symptoms through Inadequate Self, Hated Self, and Reassured Self. Unstandardized path coefficients are presented, with standard errors in parentheses. Covariates are omitted from the figure for clarity. ** *p* < 0.01; *** *p* < 0.001.

For anxiety, none of the specific indirect effects differed significantly from one another. For stress, the indirect effect through Inadequate Self was significantly stronger than the indirect effects through both Hated Self (effect = 0.08, SE = 0.02, 95% CI [0.03, 0.12]) and Reassured Self (effect = 0.06, SE = 0.03, 95% CI [0.02, 0.11]), whereas the latter two did not significantly differ from each other. The models explained 28.1% and 34.1% of the variance in anxiety and stress symptoms, respectively. Figure 1 presents the three mediation models, including the unstandardized path coefficients for the associations between postpartum body concerns, self-relating styles, and each psychological distress outcome.

Overall, these findings suggest that self-relating styles constitute key psychological processes that statistically account for the association between postpartum body concerns and psychological distress. Specifically, women experiencing greater concerns about their postpartum body also tend to respond to themselves with self-criticism and less self-reassurance, which, in turn, is associated with higher levels of depression, anxiety, and stress.

## 4. Discussion

The present study examined whether different self-relating styles, namely Inadequate Self, Hated Self, and Reassured Self, mediate the association between postpartum body concerns and symptoms of depression, anxiety, and stress in Portuguese mothers during the first two years after childbirth. The interpretation of the present findings should take into account the specific operationalization of postpartum body concerns used in this study. The composite captures two related aspects of postpartum body-related experience: current satisfaction with one’s body and concern that the body may not return to its pre-pregnancy state. Although these items showed satisfactory inter-item consistency, they do not capture the broader multidimensional nature of postpartum body image, which may include appearance evaluation, body functionality, embodiment, weight and shape concerns, and identity-related experiences. Accordingly, the present findings should be interpreted as concerning these specific postpartum body concerns rather than postpartum body image as a comprehensive construct. Plus, the relatively low mean levels of depressive, anxiety, and stress symptoms observed in the present sample are also important when interpreting the findings. DASS-21 assesses dimensional symptoms experienced during the previous week and is not a diagnostic instrument for depressive or anxiety disorders. Therefore, the observed associations should be understood as reflecting variation in psychological symptom severity within the sample rather than differences in clinically diagnosed psychopathology. The relatively low symptom levels also suggest that the findings may be more representative of psychological distress in a community postpartum sample than of women presenting with clinically significant mental health conditions. Accordingly, the observed indirect associations should not be interpreted as evidence that the identified self-relating processes explain clinically diagnosed depression, anxiety, or stress disorders.

Overall, the findings were consistent with the proposed mediation models, with self-criticism and self-reassurance showing significant indirect associations with the relationship between postpartum body concerns and psychological distress. As hypothesized, greater postpartum body concerns were associated with higher psychological symptoms. Significant indirect associations were observed for anxiety and stress, while both direct and indirect associations were observed for depression. Overall, these findings contribute to a better understanding of the psychological processes associated with maternal psychological distress during the postpartum period. Specifically, they suggest that self-relating processes may help explain why postpartum body concerns are associated with poorer maternal mental health.

The observed association between postpartum body concerns and psychological symptoms is consistent with a substantial body of evidence demonstrating that body concerns remain an important aspect of maternal adjustment well beyond childbirth. Although pregnancy and the postpartum period involve expected physiological changes, many women continue to experience dissatisfaction with their appearance, concerns about returning to their pre-pregnancy body, and perceived discrepancies between their current body and culturally valued appearance ideals. A recent systematic review and meta-analysis concluded that postpartum body dissatisfaction is consistently associated with poorer maternal mental health, particularly depressive symptoms [28]. Likewise, longitudinal evidence has shown that body dissatisfaction and negative body attitudes are prospectively associated with depressive and anxiety symptoms across the perinatal period, suggesting that body image constitutes an important vulnerability factor for maternal psychological adjustment rather than merely a concurrent correlate of psychological distress [29,30].

Our findings extend this literature by suggesting that women’s habitual ways of responding to themselves may be relevant to the association between postpartum body concerns and psychological distress. Rather than implying a direct causal pathway, the observed pattern indicates that postpartum body concerns may be particularly strongly associated with psychological distress among women who report more self-critical or less reassuring ways of relating to themselves. According to Gilbert’s evolutionary model, experiences interpreted as failures, inadequacies, or threats to social acceptance may be related to threat-protection processes, whereas self-reassurance is associated with soothing and affiliative processes that may facilitate emotional regulation [31,32]. Within the postpartum context, body changes may become particularly salient because they occur during a period characterized by substantial identity reorganization, increased social evaluation, and heightened expectations regarding motherhood. Consequently, postpartum body concerns may acquire psychological significance when they are experienced in the context of feelings of personal inadequacy or failure. However, threat-protection and soothing processes were not directly assessed in the present study, and the cross-sectional design does not allow us to determine whether these processes account for the observed associations. These theoretical frameworks should therefore be considered possible interpretations of the findings rather than demonstrated explanations.

From a clinical perspective, the findings suggest that the way women relate to themselves in the context of postpartum body concerns may be clinically relevant and warrants further investigation as a potential correlate of psychological vulnerability. Importantly, the present findings do not indicate that postpartum body concerns inevitably result in psychological distress, nor do they establish that self-relating styles cause or prevent such distress. Longitudinal and experimental research is needed to determine whether self-critical and reassuring ways of relating to oneself predict subsequent changes in psychological symptoms and whether they represent potentially modifiable processes in the postpartum period.

Although our initial hypothesis emphasised the role of Inadequate Self, all three self-relating styles significantly mediated the associations under study. Feelings of inadequacy showed larger indirect effects in the models, suggesting that perceiving oneself as “not good enough” may represent an important component of the association between postpartum body concerns and psychological distress. This finding is consistent with previous research indicating that inadequate self represents the most prevalent form of self-criticism and is strongly associated with shame, psychological distress, and body image disturbances [18,33]. Nevertheless, the significant contribution of hated self indicates that some mothers may respond to body-related concerns through more hostile forms of self-relating characterized by self-disgust, contempt, or punitive self-attitudes. Although mean levels of hated self were comparatively low in the present sample, its consistent mediating role across all three models suggests that even relatively infrequent hostile self-attitudes may be meaningfully associated with postpartum psychological distress. Together, these findings suggest that self-critical processes may represent clinically relevant vulnerability factors, helping to identify mothers who may be at increased risk of experiencing persistent psychological distress during the postpartum period.

Importantly, Reassured Self also significantly mediated the association between postpartum body concerns and all three psychological outcomes. Women reporting higher postpartum body concerns also reported less ability to respond to themselves with warmth, encouragement, and understanding, which was associated with higher levels of depression, anxiety, and stress. This finding aligns with the growing literature, suggesting that responding to body changes with acceptance and self-compassion may be associated with lower psychological consequences of appearance-related concerns, reinforcing the conceptualisation of self-reassurance as more than simply the absence of self-criticism; rather, it represents an independent protective process that is uniquely associated with psychological well-being [21,34,35]. This finding also suggests that self-reassurance should not be viewed merely as the absence of self-criticism, but as a psychological resource that may be associated with resilience and facilitate maternal adaptation to the physical and psychological challenges of the postpartum period.

Interestingly, different indirect-effect patterns emerged across psychological outcomes. For anxiety and stress, the associations with postpartum body concerns were accounted for by significant indirect effects through self-relating styles, as the corresponding direct effects were no longer statistically significant after accounting for the mediators. In contrast, for depression, both the direct and indirect effects remained significant, indicating that self-relating styles statistically accounted for part, but not all, of the association between postpartum body concerns and depressive symptoms. One possible explanation is that depressive symptoms in the postpartum period are influenced by additional associations beyond self-criticism, including broader changes in self-concept, perceived maternal competence, loss of identity, hormonal changes, sleep disruption, or reduced social support [1,2]. Previous longitudinal studies have similarly suggested that body dissatisfaction predicts later depressive symptoms even after accounting for several psychosocial factors [30], indicating that body-related experiences may exert both direct and indirect influences on depression.

In contrast, anxiety and stress appear to be more closely linked to ongoing threat monitoring and emotional regulation processes, making them particularly sensitive to individual differences in self-criticism and self-reassurance. Although this interpretation requires longitudinal confirmation, it is consistent with theoretical models positioning self-criticism as a transdiagnostic process involved in the maintenance of threat-related emotional states. These differential findings may also have clinical relevance, suggesting that intervention strategies may need to be tailored according to women’s predominant symptom profile rather than assuming that the same psychological processes operate equally across all forms of psychological distress.

These findings may also be understood within broader sociocultural models of postpartum body image [36,37]. Contemporary mothers are frequently exposed to messages emphasizing rapid recovery of the pre-pregnancy body while simultaneously navigating the physical demands of caring for an infant. Such expectations may promote body surveillance, upward social comparison, and internalization of unrealistic appearance ideals, increasing vulnerability to shame and self-critical thinking [34,38,39]. Recent qualitative work has similarly highlighted that many women perceive pressure to “bounce back” to the pre-pregnancy body, contributing to feelings of failure when body changes persist [19].

### 4.1. Clinical and Practical Implications

The present findings highlight self-criticism and self-reassurance as psychological processes that may be relevant to understanding the associations between postpartum body concerns and psychological distress. Given the growing emphasis on early identification and prevention of maternal mental health difficulties, these findings may have implications for clinical assessment and future intervention research. Specifically, they suggest that assessment of postpartum body concerns may benefit from consideration of broader self-relating tendencies, including self-criticism and self-reassurance. However, given the cross-sectional nature of the present study, these processes should not yet be considered established markers of vulnerability or causal mechanisms underlying postpartum psychological distress. Rather, the findings provide preliminary evidence that may inform further research into whether individual differences in self-relating are relevant to women’s psychological adjustment during the postpartum period.

The findings also provide a rationale for further investigation of self-criticism and self-reassurance in intervention research. The different patterns of direct and indirect effects across psychological outcomes suggest that these processes may not be similarly relevant across domains of psychological distress. While the associations between postpartum body concerns and anxiety and stress were statistically accounted for by indirect effects through self-criticism and self-reassurance, depressive symptoms remained associated with body concerns after accounting for these processes. These patterns may therefore warrant investigation in longitudinal and intervention studies examining whether changes in self-criticism and self-reassurance precede changes in postpartum anxiety, stress, and depressive symptoms, and whether targeting these processes leads to improvements in psychological outcomes.

In this context, compassion-focused and self-compassion-based interventions, which include components aimed at reducing self-criticism and fostering more compassionate self-relating, may represent potentially relevant approaches for future research [21,40,41]. However, their effectiveness for postpartum women experiencing body concerns, as well as the extent to which changes in self-criticism and self-reassurance contribute to treatment effects, remain to be established. Overall, the present findings should be considered preliminary, highlighting self-relating processes as potentially relevant areas for further investigation before solid implications for clinical practice could be framed.

### 4.2. Limitations and Future Directions

Several limitations should be considered when interpreting these findings. First, postpartum body concerns were assessed using a brief two-item composite derived from the Sociodemographic and Risk Factors Questionnaire rather than a standardized, validated postpartum-specific body image instrument. The items captured current body satisfaction and concern about persistent changes following pregnancy and showed satisfactory inter-item consistency. However, internal consistency does not establish construct validity, and the composite does not capture the broader multidimensional nature of postpartum body image. Thus, the findings should be interpreted specifically in relation to the operationalized construct of postpartum body concerns, rather than generalized to postpartum body image more broadly. Future studies should employ validated, multidimensional postpartum-specific measures.

Second, self-criticism and self-reassurance were assessed using the FSCRS, which measures general dispositional tendencies rather than responses specifically related to postpartum bodily changes. In addition, all variables were assessed using self-report measures, which may introduce common-method variance and potentially influence the observed associations. The extent of any such influence cannot be determined from the present data. Future research should consider measures specifically assessing body-related self-criticism and self-reassurance and, where feasible, multiple assessment methods.

Third, the relatively low levels of depressive, anxiety, and stress symptoms should be considered when interpreting the findings. The DASS-21 is a dimensional symptom measure rather than a diagnostic instrument; therefore, the findings reflect variation in psychological symptoms within the sample and cannot be directly generalized to women with clinically diagnosed psychiatric disorders. Replicating samples with a broader range of symptom severity and in clinical populations is warranted.

Fourth, the cross-sectional design precludes conclusions about temporal precedence or causal direction. Accordingly, the indirect effects should be interpreted as statistical associations consistent with the hypothesized mediation model rather than as evidence of causal mediation. Alternative directions of association, including the possibility that psychological distress is associated with more negative body evaluations and greater self-criticism, cannot be ruled out. Longitudinal and experimental studies are needed to clarify the directionality of these associations.

Fifth, the observed pattern differed across psychological outcomes, with significant indirect associations involving self-criticism and self-reassurance for anxiety and stress, whereas these processes accounted for only part of the association with depressive symptoms. These differences should be interpreted cautiously, particularly because the underlying theoretical processes were not directly assessed. Future research should examine additional psychological processes that may be relevant to postpartum depressive symptoms.

Sixth, participants were recruited through convenience and snowball sampling using online platforms, which may have introduced selection bias and limited the representativeness and generalizability of the findings. The relatively high proportion of mothers with higher education, living with a partner, and being primiparous may further limit generalizability to mothers with different sociodemographic and family characteristics. The eligibility criterion requiring conception within a heterosexual relationship also restricted the diversity of relationship and family configurations represented in the sample. Future studies should use more diverse recruitment strategies and include a broader range of family and relationship configurations. The postpartum duration was not included as a covariate in the original models, despite infants’ ages ranging from 2 weeks to 24 months. Because postpartum body concerns, body image, and psychological adjustment may vary across this period, differences in time since childbirth may have influenced the observed associations. The revised analyses account for postpartum duration, while longitudinal research should further examine whether these associations remain stable across different stages of postpartum recovery. Finally, data were collected between February and March 2020, coinciding with the emergence of the COVID-19 pandemic. Because pandemic-related experiences were not directly assessed, it is not possible to determine whether, or to what extent, the pandemic influenced psychological symptoms, social support, or postpartum experiences in the sample.

## 5. Conclusions

Taken together, these limitations indicate that the findings should be interpreted as evidence of associations and indirect statistical pathways within the studied sample, rather than as evidence of causal mechanisms or clinically established effects. Further research using validated multidimensional measures, longitudinal designs, more diverse samples, and multiple assessment methods is needed to determine the robustness and generalizability of these findings.

## Figures and Tables

**Table 1:** Sociodemographic and Obstetric Characteristics of the Sample.

Variable	n	%/M (SD)
Sociodemographic characteristics		
Age (years)	540	M = 32.77, SD = 5.00 (20–48)
Marital Status		
Married or cohabiting	491	90.9%
Single	32	5.9%
In a relationship, not cohabiting	11	2.0%
Separated or divorced	6	1.1%
Education		
Higher education	334	61.9%
Non-higher education	204	37.7%
Other	2	0.4%
Employment Status		
Employed	455	84.3%
Not working	85	15.7%
Monthly Household Income		
Below €1125	147	27.2%
€1125–2291	286	52.9%
€2291–3333	76	14.1%
Above €3333	31	5.7%
Infant and obstetric characteristics		
Parity		
Primiparous	343	63.5%
Multiparous	197	36.5%
Infant age (months)	540	M = 8.59, SD = 6.51 (0.5–24)
Current Parental Leave		
No	308	57.0%
Yes	232	43.0%
Complications During Pregnancy		
No complications	232	43.0%
High-risk pregnancy	205	38.0%
Maternal health complications during pregnancy	34	6.3%
Maternal health complications during/ after delivery	32	5.9%
Infant health complications after delivery	20	3.7%
Infant health complications during pregnancy	17	3.1%
Type of Delivery		
Vaginal delivery	280	51.9%
Emergency cesarean section	102	18.9%
Planned cesarean section	95	17.6%
Assisted (vacuum-assisted) delivery	108	20.0%
Gestational Age at Birth		
Term (≥37 weeks)	507	93.9%
Preterm (<37 weeks)	33	6.1%
Current Maternal Health Problems		
No health problems	436	84.3%
Has health problems	81	15.7%

Note: M = mean; SD = standard deviation. The category Employed includes employed participants and working students. Not working includes unemployed participants, homemakers, and students.

**Table 2:** Descriptive statistics and correlations between main study variables.

Variable	1	2	3	4	5	6	M (SD)
1. Postpartum body concerns	–						3.35 (1.05)
2. Inadequate Self (FSCRS)	0.26 ***	–					1.25 (0.89)
3. Hated Self (FSCRS)	0.15 ***	0.60 ***	–				0.30 (0.60)
4. Reassured Self (FSCRS)	−0.30 ***	−0.49 ***	−0.40 ***	–			1.89 (0.92)
5. Depression (DASS-21)	0.26 ***	0.58 ***	0.61 ***	−0.44 ***	–		2.41 (3.27)
6. Anxiety (DASS-21)	0.17 ***	0.41 ***	0.44 ***	−0.36 ***	0.68 ***	–	1.90 (2.79)
7. Stress (DASS-21)	0.21 ***	0.54 ***	0.43 ***	−0.38 ***	0.75 ***	0.67 ***	4.86 (4.02)

Note: FSCRS = Forms of Self-criticizing and Self-reassuring Scale; DASS-21 = Depression Anxiety Stress Scales–21. *** *p* < 0.001.

**Table 3:** Results of the parallel mediation models predicting depression, anxiety, and stress.

Effect	b	SE	*p*	95% CI
Depression R^2^ = 0.48, F (9, 530) = 53.81, *p* < 0.001				
Total Effect (c)	0.78	0.13	<0.001	[0.52, 1.04]
Direct Effect (c’)	0.27	0.10	0.010	[0.07, 0.48]
Indirect: Inadequate Self	0.22	0.06	—	[0.13, 0.35]
Indirect: Hated Self	0.17	0.06	—	[0.07, 0.33]
Indirect: Reassured Self	0.11	0.04	—	[0.05, 0.20]
Anxiety R^2^ = 0.28, F (9, 530) = 23.01, *p* < 0.001				
Total Effect (c)	0.41	0.11	<0.001	[0.19, 0.63]
Direct Effect (c’)	0.08	0.10	0.439	[−0.12, 0.29]
Indirect: Inadequate Self	0.10	0.05	—	[0.02, 0.20]
Indirect: Hated Self	0.11	0.05	—	[0.03, 0.21]
Indirect: Reassured Self	0.12	0.04	—	[0.06, 0.20]
Stress R^2^ = 0.34, F (9, 530) = 30.41, *p* < 0.001				
Total Effect (c)	0.76	0.16	<0.001	[0.44, 1.08]
Direct Effect (c’)	0.15	0.14	0.291	[−0.13, 0.44]
Indirect: Inadequate Self	0.38	0.08	—	[0.24, 0.56]
Indirect: Hated Self	0.09	0.04	—	[0.03, 0.19]
Indirect: Reassured Self	0.14	0.05	—	[0.04, 0.25]

Note: 95% CI = 95% bias-corrected bootstrap confidence interval. Significant indirect effects are indicated by confidence intervals that do not include zero.

## Data Availability

The datasets generated and/or analysed during the current study are available from the corresponding author on reasonable request.

## References

[1] Biaggi A., Conroy S., Pawlby S., Pariante C.M. (2016). Identifying the women at risk of antenatal anxiety and depression: A systematic review. J. Affect. Disord..

[2] Howard L.M., Molyneaux E., Dennis C.-L., Rochat T., Stein A., Milgrom J. (2014). Non-psychotic mental disorders in the perinatal period. Lancet.

[3] Wang Z., Liu J., Shuai H., Cai Z., Fu X., Liu Y., Xiao X., Zhang W., Krabbendam E., Liu S. (2021). Mapping global prevalence of depression among postpartum women. Transl. Psychiatry.

[4] Feldman N., Hibara A., Ye J., Macaranas A., Larkin P., Hendrix E., Aydinian T., Mittal L., Wiegartz P., Silbersweig D. (2025). Postpartum anxiety: A state-of-the-art review. Lancet Psychiatry.

[5] Ou L., Shen Q., Xiao M., Wang W., He T., Wang B. (2025). Prevalence of co-morbid anxiety and depression in pregnancy and postpartum: A systematic review and meta-analysis. Psychol. Med..

[6] Gusak N., Kendall S., Nizalova O. (2024). Understanding of perinatal mental health and its psychosocial determinants through Ukrainian women’s experience. Eur. J. Midwifery.

[7] Cash T.F., Pruzinsky T. (2002). Body Image: A Handbook of Theory, Research, and Clinical Practice.

[8] Clark A., Skouteris H., Wertheim E.H., Paxton S.J., Milgrom J. (2009). The relationship between depression and body dissatisfaction across pregnancy and the postpartum: A prospective study. J. Health Psychol..

[9] Riquin E., Lamas C., Nicolas I., Dugré-Lebigre C., Curt F., Cohen H., Legendre G., Corcos M., Godart N. (2019). A key for perinatal depression early diagnosis: The body dissatisfaction. J. Affect. Disord..

[10] Silveira M.L., Ertel K.A., Dole N., Chasan-Taber L. (2015). The role of body image in prenatal and postpartum depression: A critical review of the literature. Arch. Women’s Ment. Health.

[11] Fox B., Neiterman E. (2015). Embodied motherhood: Women’s feelings about their postpartum bodies. Gend. Soc..

[12] Di Gesto C., Preston C., Nerini A., Matera C., Grano C. (2025). Testing the tripartite influence model on body image among pregnant women. Body Image.

[13] Saxbe D.E., Rossin-Slater M., Goldenberg D. (2018). The transition to parenthood as a critical window for adult health and well-being. Am. Psychol..

[14] Ehret A.M., Joormann J., Berking M. (2015). Examining risk and resilience factors for depression: The role of self-criticism and self-compassion. Cogn. Emot..

[15] Löw C.A., Schauenburg H., Dinger U. (2020). Self-criticism and psychotherapy outcome: A systematic review and meta-analysis. Clin. Psychol. Rev..

[16] Werner A.M., Tibubos A.N., Rohrmann S., Reiss N. (2019). The clinical trait self-criticism and its relation to psychopathology: A systematic review—Update. J. Affect. Disord..

[17] Siqueira M.R.D., Limongi T.M., Salzer E.B., Bomtempo A.P.D., Meireles J.F.F., Neves C.M. (2025). Postpartum women’s body dissatisfaction: A systematic review of theoretical models and regression analyses. Int. J. Environ. Res. Public Health.

[18] Gilbert P., Clarke M., Hempel S., Miles J.N.V., Irons C. (2004). Criticizing and reassuring oneself: An exploration of forms, styles and reasons in female students. Br. J. Clin. Psychol..

[19] Christian C., Braynen A.S., Clark S.R., Donofry S.D. (2025). Body image and eating habits during pregnancy and postpartum: Themes and suggestions for maternal healthcare. J. Reprod. Infant Psychol..

[20] Spinoni M., Solorzano C.S., Grano C. (2023). A prospective study on body image disturbances during pregnancy and postpartum: The role of cognitive reappraisal. Front. Psychol..

[21] Papini N., Mason T.B., Herrmann S., Lopez N. (2022). Self-compassion and body image in pregnancy and postpartum: A randomized pilot trial of a brief self-compassion meditation intervention. Body Image.

[22] Ball N., Gilmour A., Liddelow C., Burke K.J., Lee M.F. (2026). “The pressure to do it all”: Qualitatively exploring body image, eating attitudes and mental health in postpartum women. Health Promot. J. Aust..

[23] Cohen J. (1988). Statistical Power Analysis for the Behavioral Sciences.

[24] Castilho P., Gouveia J.P. (2011). Auto-Criticismo: Estudo de validação da versão portuguesa da Escala das Formas do Auto-Criticismo e Auto-Tranquilização (FSCRS) e da Escala das Funções do Auto-Criticismo e Auto-Ataque (FSCS). Psychologica.

[25] Lovibond P.F., Lovibond S.H. (1995). The structure of negative emotional states: Comparison of the Depression Anxiety Stress Scales (DASS) with the Beck Depression and Anxiety Inventories. Behav. Res. Ther..

[26] Pais-Ribeiro J., Honrado A., Leal I. (2004). Contributo para o estudo da adaptação portuguesa das escalas de ansiedade, depressão e stress (EADS) de 21 itens de Lovibond e Lovibond. Psicol. Saúde Doenças.

[27] Hayes A.F. (2022). Introduction to Mediation, Moderation, and Conditional Process Analysis.

[28] He J., Chen X., Luo B. (2025). The association between body image and depressive symptoms in pregnant and postpartum women: A meta-analysis. Front. Public Health.

[29] Chan C.Y., Lee A.M., Koh Y.W., Lam S.K., Lee C.P., Leung K.Y., Tang C.S.K. (2020). Associations of body dissatisfaction with anxiety and depression in the pregnancy and postpartum periods: A longitudinal study. J. Affect. Disord..

[30] Hartley E., Hill B., McPhie S., Skouteris H. (2018). The associations between depressive and anxiety symptoms, body image, and weight in the first year postpartum: A rapid systematic review. J. Reprod. Infant Psychol..

[31] Gilbert P. (2009). The Compassionate Mind: A New Approach to Life’s Challenges.

[32] Gilbert P. (2010). Compassion Focused Therapy: The CBT Distinctive Features Series.

[33] Castilho P., Gouveia J.P., Duarte J. (2015). Exploring self-criticism: Confirmatory factor analysis of the FSCRS in clinical and nonclinical samples. Clin. Psychol. Psychother..

[34] Gillen M.M., Markey C.H., Rosenbaum D.L., Dunaev J.L. (2021). Breastfeeding, body image, and weight control behavior among postpartum women. Body Image.

[35] Rosenbaum D.L., Gillen M.M., Markey C.H. (2020). Feeling let down: An investigation of breastfeeding expectations, appreciation of body functionality, self-compassion, and depression symptoms. Appetite.

[36] Lee M.F., Bolton K., Madsen J., Burke K.J. (2025). A systematic review of influences and outcomes of body image in postpartum via a socioecological framework. J. Reprod. Infant Psychol..

[37] Rodgers R.F., Campagna J., Hayes G., Sharma A., Runquist E., Fiuza A., Coburn-Sanderson A., Zimmerman E., Piran N. (2024). Sociocultural pressures and body related experiences during pregnancy and the postpartum period: A qualitative study. Body Image.

[38] Lee M.F., Target C., Liddelow C., Burke K.J. (2026). Bouncing back with bliss: Nurturing body image and embracing intuitive eating in the postpartum: A cross-sectional replication study. J. Reprod. Infant Psychol..

[39] Thompson K.A., Bardone-Cone A.M. (2022). Social comparison, disordered eating, and body dissatisfaction among postpartum women. Body Image.

[40] Millard L., Wittkowski A. (2023). Compassion focused therapy for women in the perinatal period: A summary of the current literature. Front. Psychiatry.

[41] Shen M.-D., Gao R., Chen S.-B., Xu Z., Ding X.-D. (2024). The effectiveness of interventions on improving body image for pregnant and postpartum women: A systematic review of randomized clinical trials. BMC Pregnancy Childbirth.

